# An unperceived acoustic stimulus decreases reaction time to visual information in a patient with cortical deafness

**DOI:** 10.1038/s41598-020-62450-9

**Published:** 2020-04-02

**Authors:** Anthony N. Carlsen, Dana Maslovat, Kimitaka Kaga

**Affiliations:** 1 School of Human Kinetics, University of Ottawa, Ottawa, Canada; 2 School of Kinesiology, University of British Columbia, Vancouver, Canada; 3 National Institute of Sensory Organs, National Tokyo Medical Center, Tokyo, Japan

**Keywords:** Sensory processing, Neurophysiology, Stroke

## Abstract

Responding to multiple stimuli of different modalities has been shown to reduce reaction time (RT), yet many different processes can potentially contribute to multisensory response enhancement. To investigate the neural circuits involved in voluntary response initiation, an acoustic stimulus of varying intensities (80, 105, or 120 dB) was presented during a visual RT task to a patient with profound bilateral cortical deafness and an intact auditory brainstem response. Despite being unable to consciously perceive sound, RT was reliably shortened (~100 ms) on trials where the unperceived acoustic stimulus was presented, confirming the presence of multisensory response enhancement. Although the exact locus of this enhancement is unclear, these results cannot be attributed to involvement of the auditory cortex. Thus, these data provide new and compelling evidence that activation from subcortical auditory processing circuits can contribute to other cortical or subcortical areas responsible for the initiation of a response, without the need for conscious perception.

## Introduction

Responding to a stimulus with a voluntary action is typically thought to involve several processes that are mediated by cortical structures. For example, a sprinter will “get set” to launch their body out of the starting blocks, and once the starting pistol fires the sprinter must recognize that the signal has occurred and initiate that planned response. The processes involved in planning a response (preparation) and holding it ready until the go-signal occurs (storage) have been ascribed principally to cortical motor and premotor areas^1–3^. Similarly, processes involved in stimulus recognition (detection/discrimination) and response initiation are thought to occur in cortical sensory and association areas as well as frontal cortical regions^4^.

However, data from a paradigm involving the presentation of an unpredictable high-intensity auditory stimulus that is capable of eliciting a startle reflex has suggested that lower *subcortical* structures may play a role in response initiation processes^5^. Typical mean reaction time (RT) to the onset of an auditory stimulus is approximately 140–150 ms, with reaction to a visual stimulus being slightly longer at 180–200 ms^6^. Yet, when a loud startling acoustic stimulus (SAS) is presented, RT is dramatically shortened to approximately 70–80 ms on average, while the characteristic features of the response remain largely unchanged^7,8^. The startle reflex is primarily mediated by brainstem nuclei^9^ and elicits a pattern of generalized flexion in various muscles with latencies of 50–100 ms. Given the short latency of both the startle reflex and prepared response, early explanations of RT speeding by startle were that the “voluntary” reaction was too short to have involved the normal cortically mediated stimulus-response processing pathways and was somehow driven at the speed of the startle response. In order for this to occur, it was argued that storage and/or initiation of the response must have involved subcortical structures common to both the startle and voluntary response channels^7^. This notion of subcortical involvement has also been tested in patients with hereditary spastic paraplegia, whose corticospinal tracts have been severely damaged resulting in substantial RT delays. Presenting a SAS led to RT shortening in these patients similar to that seen in healthy controls, suggesting that the startle-elicited response output somehow bypassed the damaged corticospinal pathways^10^.

While the presentation of a startling stimulus is thought to decrease response latencies through the involvement of subcortical pathways, the presentation of a non-startling auditory stimulus in conjunction with a visual stimulus can also result in shorter RTs. This so-called “redundant signal effect” occurs when stimuli of different modalities are presented, and a variety of models have been proposed to explain the RT reduction including statistical facilitation, multisensory integration, and crossmodal attention; see reviews^11–13^. Each viewpoint outlines different mechanisms responsible for shortened response latency, which also depend on the relative stimulus locations and timing of presentation^14,15^. The integration of auditory and visual information is thought to involve a variety of cortical and subcortical structures including unisensory cortices and superior temporal cortex^16,17^, as well as reticular formation and superior colliculus^18,19^. However, the relative contributions of these structures during multisensory integration is still a matter of debate^20^, as is the degree of cortical versus subcortical involvement in response initiation processes.

**Figure 1:**
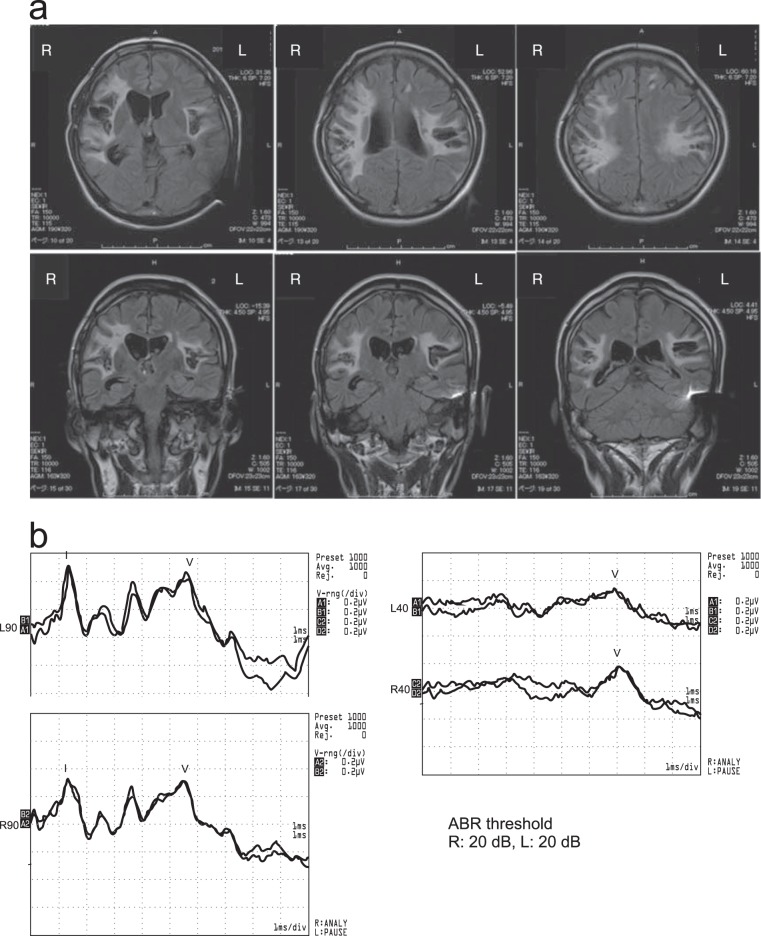
Details for patient exhibiting complete bilateral cortical deafness. Panel (a) shows an anatomical MRI of the patient showing bilateral lesions to auditory cortices, radiations, and postcentral gyruses. Panel (b) shows an intact auditory brainstem response (ABR) with thresholds of 20 dB in both ears. Additional details are provided in the Methods section. See Kaga *et al*.^21^ for original images and a more detailed patient description and testing protocols. Reproduced with permission.

In order to investigate the involvement of subcortical circuits in response initiation, we carried out a simple RT experiment in a patient with bilateral cortical deafness, in which an auditory stimulus of varying intensities was presented in conjunction with a visual go-signal. Complete bilateral cortical deafness is an exceedingly rare condition, and the patient reports no conscious perception of sound, a result of cortical auditory damage following a bilateral stroke^21^ (Fig. 1a). However, he exhibits a normal auditory brainstem response (ABR, see Fig. 1b), indicating that secondary pathways are intact. In our experiment, the patient was required to make a wrist extension movement as quickly as possible in response to the onset of a visual go-signal. On some trials the visual stimulus was accompanied by an acoustic stimulus of variable intensity: Quiet (80 dB), loud (105 dB), very loud (120 dB). The stimulus was presented either concurrent with, or 200 ms prior to the visual signal. Our primary research question was whether the presence of an unperceived auditory stimulus would result in RT facilitation. If the patient exhibited a startle reflex and accompanying reduction in RT when presented with the very loud (120 dB) acoustic stimulus, this result would be attributed to involvement of startle reflex circuitry in the accelerated initiation of a prepared response, due to the intact subcortical auditory circuits. Alternatively, if no startle reflex was observed, yet a reduction in RT was nevertheless found when presented with an acoustic stimulus (irrespective of intensity), this result would be indicative of multisensory response enhancement occurring in the absence of awareness of auditory input.

## Results

### Reaction time

**Figure 2:**
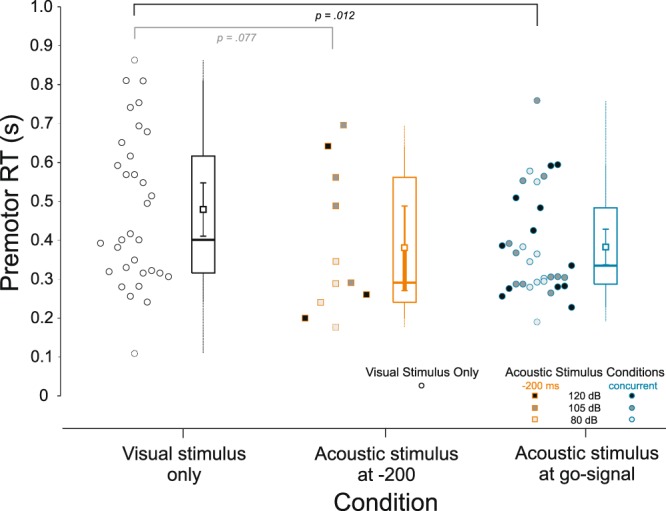
Premotor reaction time (RT) by condition: Boxplots and individual trial data. Unfilled circles show RTs for trials with the visual stimulus alone. Filled symbols show RTs for trials where the acoustic stimulus was presented simultaneous with the visual stimulus (blue outline circles), and 200 ms prior to it (orange outline squares). Box boundaries show the median as well as 1st and 3rd quartiles. Whiskers show the range of the data (dotted = untrimmed, solid = top/bottom values trimmed). The small squares inside each boxplot show the mean with 95% confidence intervals.

Even though the patient reported a complete lack of awareness of any acoustic stimulus, results show that regardless of stimulus intensity, an *unperceived* acoustic stimulus presented concurrent with the visual go-signal led to a reliable and substantial (~100 ms) reduction in RT (Fig. 2). Although single-participant designs are often descriptive in nature, the present results were statistically verified using non-parametric randomization tests (see Methods). First, randomization tests compared RTs obtained when the three intensities of acoustic stimuli (80, 105, and 120 dB) were presented simultaneous with the visual go-signal. These tests showed no statistically reliable difference between stimulus intensities (all p-values > 0.442). Similar results were obtained for trials where the acoustic stimulus was presented 200 ms prior to the visual go-signal (all p-values > 0.701). This is exemplified in Fig. 2 where individual trial premotor RT for all conditions is plotted alongside grouped descriptive statistics. Thus, for randomization tests comparing acoustic stimulus trial RTs to visual-only trials, RTs were collapsed for the acoustic stimulus conditions presented simultaneous with the visual go-signal, as well as for the acoustic stimulus presented 200 ms prior to the visual go-signal. This analysis confirmed that when an auditory stimulus was presented simultaneous with the visual stimulus, mean RT was reliably shorter (*p* = 0.012) even though no differences existed between intensities. A Bayesian t-test also corroborated this finding, providing moderate evidence that the data were ~4.6 times more likely under the alternative hypothesis that the stimulus led to shorter RTs (BF_+0_ = 4.567). When the acoustic stimulus was presented 200 ms prior to the visual go, RT was also shortened by ~100 ms on average; however, this result did not reach statistical significance (*p* = 0.077), and would be considered inconclusive with respect to the null hypothesis using Bayesian inference (BF_+0_ = 1.312).

**Figure 3:**
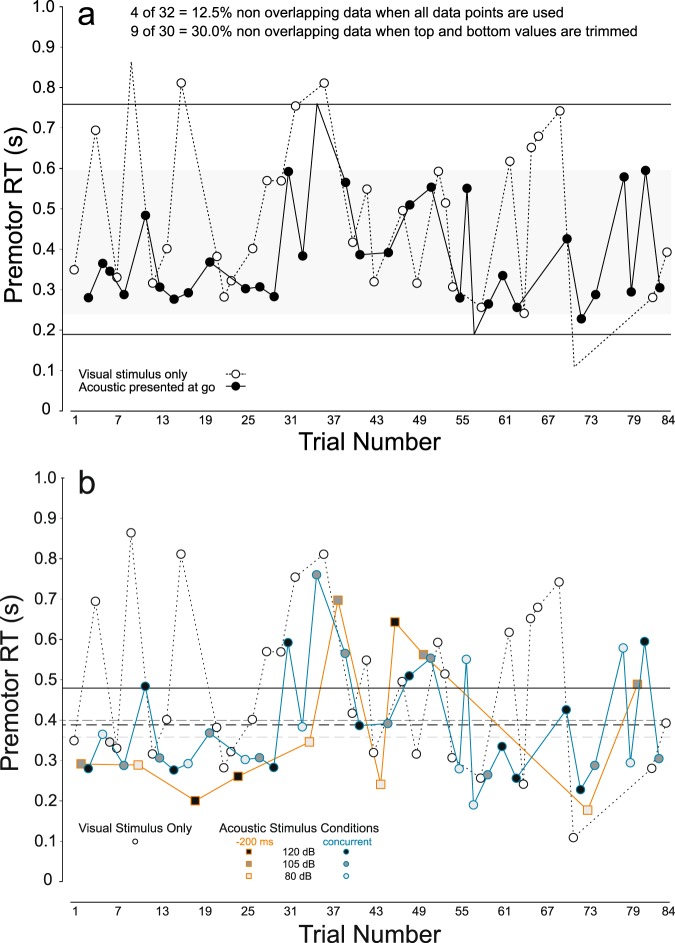
Trial-by-trial reaction time (RT) data. Panel (a) shows percentage of non-overlapping data (PND) where premotor RT in non-error trials is shown in order of presentation where the visual stimulus was presented alone (unfilled circles), or where an acoustic stimulus was presented simultaneous with the visual go. Black horizontal lines represent the upper and lower extents of overlapping data points between the conditions when all data is taken into account, resulting in a PND of 12.5%. When the top and bottom values in each condition are trimmed (missing markers) PND is 30% (grey shaded area). Panel (b) shows premotor reaction time (RT) observed in all non-error trials in order of presentation. Unfilled circles (black outline) show RTs for trials where the visual stimulus (VS) was presented alone, filled circles (blue outline) show RTs for trials where the acoustic stimulus (AS) was presented simultaneous with the visual stimulus, and filled squares (orange outline) show RTs for trials there the AS was presented 200 ms prior to the VS. Black filled symbols represent the 120 dB AS, dark grey symbols represent a 105 dB AS, and light grey symbols represent an 80 dB AS. Solid horizontal line shows mean RT for VS only, and dashed lines shows mean RT in conditions where the AS was presented simultaneous with the VS (black = 120 dB, dark grey = 105 dB, light grey = 80 dB).

Percentage of non-overlapping data (PND) is presented in Fig. 3a as a surrogate for effect size^22^ for the visual stimulus only (unfilled circles) as compared when the auditory stimulus was presented simultaneous with the visual stimulus (filled circles). When all obtained RT values in these conditions are considered, only 4 of 32 (12.5%) values are non-overlapping (see values outside of horizontal bars, Fig. 3a). However, one limitation of PND is that it may erroneously represent effects when outliers are present in the data^23^. When the top and bottom values of each distribution are trimmed, 9 of 30 (30.0%) of values are non-overlapping (see values shown outside shaded area, Fig. 3a), which may be considered as a small effect^24^.

Trial-by-trial RT is presented in Fig. 3b to accurately depict RT trends as they evolved over time for the visual stimulus only condition (unfilled circles) as compared to all of the auditory stimulus conditions (different filled symbols), and as a function of whether the auditory stimulus occurred simultaneous with the visual stimulus (filled circles), or 200 ms prior to it (filled squares). While the mean RTs (horizontal lines) clearly indicate that reactions were faster when an acoustic stimulus was presented (dashed lines) as compared to when the visual stimulus was presented alone (solid line), this is also supported by the individual trial data.

### Startle reflex indicators

**Figure 4:**
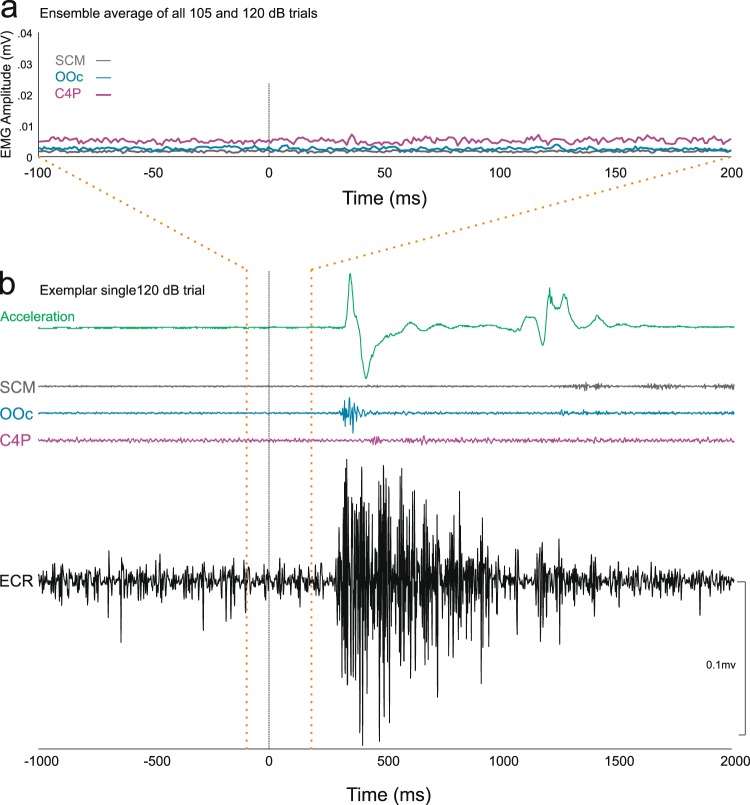
Overall startle reflex and single trial data from a patient with cortical deafness. Panel (a) shows ensemble average of raw rectified EMG from startle reflex indicator muscles sternocleidomastoid (SCM), orbicularis oculi (OOc) and C4 paraspinal (C4P) time locked to acoustic stimulus onset (vertical line) for all trials where a 105 or 120 dB stimulus was presented. Note that time scale shows from 100 ms prior to the acoustic stimulus until 200 ms following onset. Panel (b) shows single trial accelerometer data as well as raw EMG from SCM, OOc, C4P, and extensor carpi radialis (ECR) where a 120 dB acoustic stimulus was presented concurrent with the visual go-signal. Solid vertical line indicates stimulus onset. Note that time scale shows from 1000 ms prior to stimulus until 2000 ms following onset. Dashed vertical lines show the time window in B that was used to calculate A.

Although our primary finding is that RT facilitation due to an acoustic stimulus can occur without conscious perception of the stimulus, a secondary result was that a startle reflex was *not* observed following presentation of the auditory stimuli, irrespective of intensity. Specifically, no activity was observed in the muscles typically associated with a startle reflex in the 200 ms following the onset of the 120 dB auditory stimulus^25^, including orbicularis oculi (OOc), sternocleidomastoid (SCM), and C4 paraspinal (C4P) (Fig. 4). Although we expected to observe a startle and/or blink reflex due to the intact auditory brainstem response, some previous studies have reported the startle reflex was also abolished in patients who suffered lesions to the temporal lobes and auditory cortices^26,27^. The absence of startle reflex activity in this patient, even following first presentation of the loudest stimulus, may be directly related to the loss of input from auditory cortical areas. It is possible that higher structures may act in a top-down manner to supress the startle reflex even though the brainstem circuits are intact. As such, the lack of startle reflex observed here seems to represent top-down chronic sensory gating of these brainstem mediated responses and may represent a cortically-mediated neuroplastic adaptation to prevent unwanted reactions to unperceived stimuli.

## Discussion

The primary finding from the present experiment is that RT was significantly (~100 ms) shorter in the presence of a concurrent auditory stimulus (Fig. 2), despite the participant being unable to consciously process sound^21^. This novel result indicates that secondary auditory pathways can contribute to the processes involved in response initiation, even when the auditory stimulus is not perceived. These pathways appear to be activated at all auditory intensities presented in the current study (80, 105, & 120 dB) and the data provide compelling evidence that activation solely from subcortical auditory processing circuits can contribute to other cortical or subcortical areas responsible for the initiation of a response. The reduction in RT associated with the presentation of both an auditory and visual stimulus has previously been ascribed to many different mechanisms, including statistical facilitation, multisensory integration, and crossmodal attention^11–13^. Although our results cannot definitively support one particular explanation, they strongly implicate a subcortically mediated pathway that does not involve primary auditory cortex in the production of the faster responses, and indicate that multisensory response enhancement can occur without overt perception of the stimulus.

The initiation of a response requires a number of processes including preparation of the motor commands, identification of the go-signal, and an increase in activation in the initiation-related circuits in order to release the prepared motor commands. Although concurrent presentation of a tone and visual target has been shown to enhance response preparation in a choice RT task^28^, the current study employed a simple RT paradigm, in which preparation of the response would be expected to occur in advance of the go-signal, and thus faster response preparation is an unlikely candidate for the observed RT facilitation. In terms of identification of the go-signal, perceptual processing of multiple stimuli has been shown to be affected by both multisensory integration and crossmodal spatial attention, although the relative contribution of these effects depends on the relative timing between stimuli^13^. Crossmodal attention is considered largely a bottom-up/automatic process^29,30^ and thus could occur even without conscious perception of the auditory stimulus. However, the benefits of crossmodal attention shifts are typically maximized when the auditory stimulus precedes the visual stimulus by 50–300 ms^31,32^, yet in the current study the RT facilitation only reached statistical significance when the two stimuli were presented concurrently. Instead, concurrent stimuli are thought to promote multisensory integration, whereby sensory information is processed more efficiently^33,34^, which may have contributed to the faster RT observed when the auditory stimulus was presented.

While the observed response latency may have been affected by a faster identification of the go-signal, the data indicate it is unlikely that RT facilitation is *solely* due to these perceptual/sensory processes. It is well documented that RT is shorter with increasing stimulus intensity, an effect attributed to faster sensory processing^35^, yet in the present experiment there was no differential effect of stimulus intensity (80 dB, 105 dB, or 120 dB) on the RT effect observed (Fig. 2). Once the go-signal is identified, neural activation in initiation-related circuitry still needs to be increased to above an “ignition point” or threshold that results in the release of the prepared motor commands^4,36^. This initiation process has shown to be sped up by multiple stimuli, due to additive contributions associated with each individual stimulus^37,38^. In this co-activation model, each stimulus provides additive activation to the thalamus, which receives input from both visual and auditory stimuli^39^ and has been previously implicated in response initiation^40^. Thus, in co-activation, as well as in drift diffusion models^41^, redundant signals provide a faster rate of activation accumulation, resulting in a shorter time required to reach initiation threshold. Interestingly, in a previous investigation of this additive initiation model, when an 80 dB auditory cue was presented concurrently with a visual go-signal, the RT facilitation was 93 ms^37^, similar in magnitude to that seen in the current experiment. Furthermore, this multisensory RT reduction could not be explained by a statistical facilitation model, whereby the faster reaction is due to a race between processing of the independent stimuli^42,43^. In the current study, we also believe statistical facilitation to be an unlikely explanation, as the racehorse model requires a response to each stimulus, which independently race to reach a response threshold. Although it is conceivable that an unperceived stimulus could cause a response via subcortical circuitry, the lack of response to the auditory stimulus when presented 200 ms prior to the visual go-signal suggests this did not occur in the present experiment. On these trials, the patient did not respond to the auditory stimulus, as this would likely have resulted in a RT reduction of ~200 ms compared to the condition when the auditory cue was presented simultaneously with the visual stimulus; yet RTs for both auditory conditions were similar to each other (and both ~100 ms faster than visual stimulus-only RTs). Similarly, it does not appear that involuntary response triggering by the startling stimulus is a plausible explanation for the results, the patient did not exhibit a startle reflex (Fig. 4) and showed a similar RT reduction for all stimulus intensities.

Thus, the current results may be attributable to a variety of processes involved in multisensory response enhancement, including both faster identification of the go-signal and less time to reach the threshold for the initiation of the motor commands. While the exact locus of the RT facilitation effect is unknown, the lack of awareness of the auditory stimulus by the patient clearly implicates unconscious stimulus processing in subcortical and secondary auditory pathways, which contribute activation to voluntary response initiation circuits and thus shorter RTs. When considering other models of hierarchical multisensory processing, it is typically posited that information from different sensory modalities is transmitted to their respective primary sensory cortices via separate channels before being combined in various sensory integration centres^44–46^. However, some studies have shown that multi-modality processing can occur in cortical regions thought to be unisensory in nature^16,47^. This multi-modality activation is thought to result from cortico-cortical activation, but in the present patient this is unlikely to be the case due to extensive damage to the auditory cortices. This is not to say that the primary auditory cortex does not contribute to multisensory integration in non-patient populations. Indeed, studies involving animal models have shown that cryogenic deactivation of the auditory cortex modulates multisensory integration in the superior colliculus^48,49^, and other findings have demonstrated multisensory contributions in low level cortical structures that were previously thought to be unisensory in nature; see review^16^. However, it does appear that many brain structures are involved in perceptual processing and activation from subcortical auditory processing circuits can contribute to other cortical or subcortical areas responsible for sensory integration and response initiation. We next turn our attention to the possible structures and pathways that may contribute to the observed decrease in response latency.

**Figure 5:**
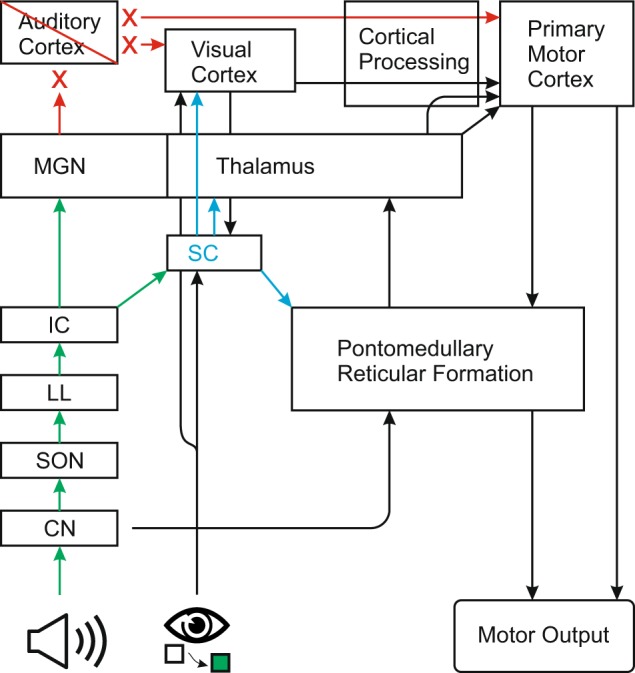
Schematic model of proposed neural pathways underlying the described RT shortening. Green arrows show connections between structures involving acoustic stimuli. Blue arrows show primary outputs from superior colliculus. Red items indicate disrupted pathways and structures. Speaker icon represents acoustic stimulus; eye and squares icon represents visual stimulus. CN = cochlear nucleus; IC = inferior colliculus; LL = lateral lemniscus; MGN = medial geniculate nucleus; SC = superior colliculus; SON = superior olivary nucleus.

The subcortical structures involved in providing this additional activation may either be part of the primary auditory circuit or secondary auditory pathways (Fig. 5). The primary auditory pathway leads from the cochlear nuclei to the superior olivary nuclei, which are concerned with sound localization. From there, axons join others from the cochlear nuclei in an ascending pathway, where some axons synapse in the nuclei of the lateral lemniscus, at the level of the pons. All ascending fibres passing through the lateral lemniscus synapse at the inferior colliculus, at the level of the midbrain. Postsynaptic cells of the inferior colliculus project to the medial geniculate nucleus of the thalamus with neurons from the geniculate body terminating in primary auditory cortex^50^. Structures in the primary auditory pathway appear to primarily integrate auditory and somatosensory information^51,52^, whereas secondary auditory pathways (through reticular formation and superior colliculus), provide information that is integrated with vision^18,19^. Indeed, it has been demonstrated that activity in superior colliculus is increased when visual and auditory stimuli are combined, and that many of the neurons that exhibited changes in firing following multisensory input had descending projections to motor and premotor areas in brainstem and spinal cord^18^. Furthermore, increased activity in superior colliculus has been associated with shorter latency onset of saccades^19^, as well as covert visual processing in hemianopic patients^53^. Thus, the present data point to the possibility that an acoustic stimulus could act to increase premotor activation in subcortical structures including thalamus, brainstem, and spinal cord, thereby “priming” the motor output system for upcoming descending efferent drive. Alternatively, higher structures implicated in secondary auditory pathways also project directly to motor thalamus and primary visual cortex. Because thalamus has been strongly implicated in providing activation leading to the initiation of motor responses^40^, inputs from subcortical auditory circuits may directly impact response initiation processing. For the current patient, the intact auditory brainstem response suggests that input from the cochlea is transferred at least to the level of the inferior colliculus and/or medial geniculate nucleus^54,55^. Thus, lower level structures involved in secondary auditory circuits likely contributed to the faster RTs observed in this patient following the presentation of an unperceived acoustic stimulus, via increases in response-related activity in cortical and subcortical structures. These data show that secondary auditory pathways and centres associated with stimulus integration are a potential mechanism by which the response speeding occurred. Greater knowledge of the neural circuitry associated with sensory processing and response initiation may ultimately lead to a better understanding of how human movements are generated and significant advances in rehabilitation of patients with disorders involving sensory perception^56^, movement initiation, and movement execution.

## Methods

### Patient

One male participant (Age = 40 years) with complete bilateral cortical deafness participated in this case study. His condition has been thoroughly described previously^21^, but in short, as the result of a 2007 rupture of the right internal carotid-posterior communicating artery and a subarachnoidal hemorrhage, the patient suffered extensive infarction of both left and right primary auditory cortices and auditory radiations (Fig. 1a). While other localized damage occurred resulting in the loss of some motor function, this was recovered completely; however, complete loss of hearing and some somatosensory and vestibular function did not recover at all. Pure tone audiometry showed thresholds that were “off the scale” (i.e., no perception even at the highest tested intensity) and speech audiometry showed 0% speech discrimination^21^. The patient reports no sensation or perception of sound whatsoever; however, audiometry showed a normal ABR with thresholds of 20 dB in both ears (Fig. 1b). Prior to testing he completed a written assessment of hearing, reporting 0% sensation in all sound categories including perception of sound, noise, sound of breathing, heartbeat, sneezing, growling stomach, or vibration sounds. Otherwise, the patient has shown normal reading, writing, and intellectual abilities, with only a minor visual memory impairment. The patient provided written informed consent prior to beginning the experiment, and testing occurred at the National Tokyo Medical Centre: Otorhinolaryngology Outpatient Clinic, Tokyo, Japan. Following the experiment, the patient was debriefed and reported being unaware that auditory stimuli were presented during the testing protocol. The procedure was approved by members of the National Tokyo Medical Centre ethics review board and followed all of its guidelines and regulations.

### Apparatus and task

During the experiment the patient sat in a chair in front of a desk with his right arm resting comfortably on the desktop approximately parallel to the body midline with the ventral forearm and palm down. A laptop computer (Dell XPS13) was placed approximately 1 m in front of the patient. The task for the patient was to perform a simple right wrist extension movement as quickly as possible upon appearance of a green square “go” signal on the laptop screen. Although the movement was untargeted, the patient was instructed to use only a wrist movement to perform the reaction time (RT) task and to not lift the entire arm. Each trial began with a visual warning signal “準備します” (“prepare”) displayed on the computer screen for 1 s along with a black outline (2 mm wide) of a 3.4 cm square displayed against a grey background. The warning text then disappeared which was followed by a 2–2.5 s variable foreperiod before the square turned green. The patient then reacted as quickly as possible with a right wrist extension. RT feedback was then provided for 3.5 s before the sequence started again.

### Procedure

The patient performed 20 practice trials where only the visual stimulus was presented. This was followed by 4 blocks of 21 testing trials. Each block was made up of 9 control (visual stimulus only) trials, 9 trials where a 25 ms duration broadband acoustic stimulus (equal power from 1 Hz to 22 kHz) was presented concurrent with the onset of the visual stimulus (3 @ 80 dB, 3 @ 105 dB, and 3 @ 120 dB), and 3 trials where the acoustic stimulus preceded the visual stimulus by 200 ms (1 @ 80 dB, 1 @ 105 dB, and 1 @ 120 dB). Different stimulus intensities were chosen as they would be expected to result in a different proportion of trials in which a reflexive startle response would be observed. Previous research has shown that presentation of a 80 dB, 105 dB and 120 dB result in elicitation of a startle reflex on approximately 0%, 50% and >80% of trials, respectively^57,58^. Trials in which the acoustic stimulus preceded the visual stimulus were included such that if the response was triggered involuntarily, latencies would clearly show that response initiation was due to the acoustic rather than the visual stimulus. In addition, a presentation of the acoustic stimulus 200 ms prior to the visual stimulus was chosen because at this time point, the participant's preparation level would be expected to be at a high and consistent level^59^. Trial conditions were randomized within each block and the entire testing procedure comprised 84 RT trials. The acoustic stimulus was generated using a custom LabView program (National Instruments Inc.) and output as an analog signal, amplified (Lepai LP-2020TI), and presented via a loudspeaker (MG Electronics M58-H) placed 30 cm directly behind the head of the patient. Sound level intensity was verified using a precision sound level meter (Casella CEL-254, A-weighting, impulse setting).

### Recording equipment

Surface electromyographic (EMG) data were collected from the muscle bellies of the right extensor carpi radialis longus (ECR; agonist), right flexor carpi radialis (FCR; antagonist), as well as traditional startle indicator muscles including the left orbicularis oculi (OOc), left sternocleidomastoid (SCM), and left C4 paraspinal (C4P) using bipolar preamplified (gain = 10) surface electrodes (DE-2.1, Delsys Inc.), attached to an external amplifier (Delsys Bagnoli-8). The electrode sites were prepared by lightly scrubbing and wiping with alcohol swabs to decrease electrical impedance. A reference electrode (4 ×4 cm, tens electrode, Lemonbest Inc.) was attached to the right lateral epicondyle. To capture displacement onset, a single axis piezoelectric accelerometer (Type 4375, Brüel & Kjær) was attached to the middle of the back of the patient's hand (proximal to the third metacarpophalangeal joint) using tape. The accelerometer was connected through an inline charge amplifier (Type 422, PCB Electronics) to a power supply and coupler (Model 5114, Kistler Inc.). Raw bandpassed (20–450 Hz) EMG and raw accelerometer data were digitally sampled at 1000 Hz for 3 s using a USB data acquisition unit (USB-6001, National Instruments Inc.). For each trial, data acquisition began 1 s prior to appearance of the visual go-signal, and was stored for later analysis.

### Data analysis

Practice trials were not included in analysis. Five testing trials were removed from analysis because the patient reacted prior to the visual or acoustic stimulus, and were considered to be anticipation. Three trials were removed for slow responses (RT > 1 s). This resulted in an inclusion rate of 90.5%. EMG burst onsets were identified for all muscles on each trial using a thresholding algorithm that identified the point at which EMG activity began a sustained increase above baseline. The temporal location of his point was determined as the first point where the baseline corrected, rectified and filtered (25 Hz low-pass elliptic filter) EMG signal increased more than 2 standard deviations above the level of activity observed in baseline (mean of the first 500 ms of data collection and prior to any stimulus onset), and remained elevated for at least 25 ms. This point was visually confirmed and corrected if necessary^60^. Reaction time (RT) was defined as the time of EMG onset in the ECR muscle with respect to the visual go-signal. The occurrence of a startle reflex was defined as an onset of EMG occurring in either the OOc, SCM, or C4P within 200 ms of presentation of the auditory stimulus. A comparatively large startle reflex onset window^25^ was used because a delayed startle reflex has been previously observed in some patient populations^61^.

RT data were assessed using visual analysis techniques, and trial-by-trial descriptive statistics are presented in Fig. 3 including percentage of non-overlapping data, a measure of effect size for single case experiments, whereby a smaller amount of overlap (higher amount non-overlapping data) indicates a larger effect of the intervention or treatment^24^. RT data were then subjected to permutation (randomization) analyses, as this type of test is valid for single case experiments because there are no underlying distribution assumptions^23^. The randomization procedure was carried out using R software^62^ with the single case data analysis (SCDA) plugin^63^ using a 1000 iteration Monte Carlo randomization. In brief, the observed RT values are randomly reordered while the order of the independent variable (trial type/condition) is held constant. The difference between the obtained condition means based on the new randomization is then determined. This procedure is carried out N times (1000 in the present case), and the number of randomly re-ordered data sets where the mean difference exceeds the actual observed mean difference is determined. The *p* value of the randomization test is the proportion of randomizations that exceed the observed difference^23^. That is, if this number is less than 5%, it can be concluded that the observed difference is unlikely to have occurred due to chance^63^. Initial tests were carried out comparing the different intensity acoustic stimulus trials to test if RT differences existed between intensities (80, 105, 120 dB). This procedure was completed for trials where the acoustic stimulus was presented simultaneous with, and 200 ms prior to the visual go-signal. No differences were detected between intensities (see Results), therefore, the acoustic stimulus trial data were collapsed across intensities and randomization tests were carried out comparing trials where an acoustic stimulus was presented versus those with only the visual stimulus. This was done separately for trials where the acoustic stimulus was presented concurrent with the visual go, or presented 200 ms prior to it. In order to further examine any differences in RT seen between conditions, Bayesian statistical analysis was used to test the likelihood that these results fit the alternative hypothesis that presentation of an acoustic stimulus led to shorter RTs. As such, separate Bayesian t-tests were performed comparing using JASP software (version 0.9) to estimate Bayes factors for comparisons between trials with the visual stimulus alone and trials where the acoustic stimulus was presented concurrent with the visual go, or presented 200 ms prior to it. All acoustic stimulus intensities were grouped for each analysis.

## Data Availability

All data generated or analysed during this study are included in this published article.
